# Group randomized trial of teaching tobacco-cessation counseling to senior medical students: a peer role-play module versus a standardized patient module

**DOI:** 10.1186/s12909-019-1668-x

**Published:** 2019-06-25

**Authors:** Kye-Yeung Park, Hoon-Ki Park, Hwan-Sik Hwang

**Affiliations:** Department of Family Medicine, Hanyang University College of Medicine, 222, Wangsimni-ro, Seongdong-gu, Seoul, 04763 South Korea

**Keywords:** Undergraduate medical education, Role playing, Standardized patient, Tobacco cessation counseling, Objective structured clinical examination

## Abstract

**Background:**

An important barrier to smoking-cessation counseling for physicians is a lack of education at the undergraduate level. Interactive methods such as peer role-play (RP) or modules utilizing standardized patients (SPs) may be effective for medical students to enhance their performance on tobacco cessation counseling. This study compared the effectiveness of a module using SPs to that of a RP module for undergraduate medical students on tobacco cessation counseling.

**Methods:**

This study was conducted over a single week of the family medicine clerkship. One hundred and thirteen fourth-year medical students were randomized into either the SP group or the RP group. A RP module involved a ten-minute encounter between the student doctor and the student patient followed by five minutes of feedback from the observer student using a group developed checklist. In a SP module, each student was asked to interview a SP portraying a smoker with willingness to quit. After the encounter, the SP provided five minutes of direct oral feedback to the student. In both modules, the total intervention lasted three-and-half hours and was supervised by faculty staff. Students’ objective structured clinical examination (OSCE) scores were evaluated to determine their tobacco cessation counseling skills. Four evaluation periods were conducted at baseline, postintervention, post-clerkship, and before receiving the Korean medical licensing examination (KMLE). Students’ smoking knowledge test scores and counseling self-confidence levels at pre- and post-intervention were also compared.

**Results:**

In both groups, post-intervention OSCE scores increased significantly compared to baseline (Cohen’s *d* 0.87, *p* < 0.001 in SP group; *d* 0.77, *p* < 0.001 in RP group). However, there were no differences between the two groups. Students achieved the highest OSCE score for smoking-cessation counseling before the KMLE. After training, student self-confidence and smoking-knowledge test scores increased significantly, regardless of the type of module. Self-confidence was higher in the SP group compared with the RP group (*d* 0.37, *p* = 0.01).

**Conclusions:**

Peer role-play may be equivalent to the SP method with regard to knowledge and skills reported during smoking-cessation counseling and SP method may be better in self-confidence. Cost and student self-confidence may be important factors when choosing among the teaching methods for smoking-cessation counseling.

## Background

Smoking is one of the most common preventable causes of death worldwide [1, 2]. In Korea in 2010, 21.3% of male deaths among all ages occurred due to smoking [3]. Although smoking rates have decreased significantly in many developed countries, Korea’s male smoking rate remains higher (31.4%) than the average (23.9%) for the 34 member countries of the Organization for Economic Cooperation and Development (OECD) [4, 5]. Based on large-scale population surveys in Korea in 2016, 30.4% of men in their thirties and 25.0% of men in their forties were smokers [5].

About 70% of smokers report that they would like to quit smoking, but fewer than one in 10 smokers achieved cessation success [6]. While a variety of smoking-cessation aids, such as counseling and pharmacological interventions, are available, two-thirds of smokers use no treatment when trying to quit [6, 7]. Many studies have determined that brief and simple advice about quitting smoking from healthcare professionals can make a difference in quit rates [8, 9]. A systematic review of 17 studies revealed a significant increase in quit rates by a further 1 to 3% following brief advice to smokers compared to no advice or routine care [10]. Additionally, concurrent use of behavioral counseling interventions and pharmacologic support during smoking cessation can double the rate of successful abstinence [11]. However, medical practitioners raise the subject of smoking with only a small proportion of their smoking patients, even though they are in a uniquely strong position to assist their patients in smoking cessation [12]. The National Health Interview Survey in U.S. has revealed 53% of workers were not advised to quit smoking by their healthcare providers [13]. A multi-center study in European countries has also shown that about 30% of general practitioners did not enquire about smoking unless the patient had smoking-related symptoms, though many of them perceived the discussion of smoking cessation as part of their job [14]. The most important barrier for physicians in smoking-cessation interventions is a lack of education at the undergraduate level [15–17]. There is a growing awareness of the necessity for tobacco education for undergraduate medical students and physicians and its important role in improving public health [18–20].

There are several methods for teaching smoking-cessation skills to medical students, including role-play, seminars, and rehearsal with simulated patients, and these methods can be combined with several feedback methods, including audiotape feedback, videotape feedback, mutual peer feedback, and standardized patients’ (SPs) direct oral feedback [20–22]. Many studies have demonstrated the effectiveness of smoking-cessation teaching methods. Results suggest that interactive methods, such as peer role-play or modules utilizing SPs, may be effective at enhancing knowledge and belief about tobacco-dependence treatment for undergraduate medical students compared with lecture-style methods [20, 22]. Communication-skills-oriented educational methods were shown to affect students’ clinical skills and behaviors regarding smoking-cessation counseling. One study compared two interactive modules—one involving SPs and one with peer role-play—for training communication skills to nursing students, and found that the SP-based method was superior to peer role-playing [21]. Another study suggested that role playing provided students with a high level of smoking cessation counseling skills similar to those provided by practicing with SPs [23].

Although two educational modules involving SPs and peer role-play are widely used for teaching patient counseling, there is a lack of evidence from comparisons between the two methods for use in smoking-cessation education. The cost of an SP smoking-cessation practice exercise is more expensive than that of a role-play. Beyond the costs for administrative staff, which is necessary in both methods during the exercise, SP method further costs for training the SPs and payment of salaries [23]. One study comparing the effectiveness of two modules on teaching motivational interviewing found no difference between two modules, in which the primary outcome was the quality of interview [24]. We aimed to compare the effectiveness of a module involving SPs in a one-to-one communication module with direct oral feedback from the SP versus peer role-play with mutual feedback for undergraduate medical students on tobacco cessation counseling. Our study specifically aimed to evaluate the two modules by measuring scores from objective structured clinical examination (OSCE) on tobacco-cessation counseling, students’ self-reported questionnaires on self-confidence, written-knowledge test scores on tobacco cessation, and short-term and long-term retention effects after education from one of the two modules.

## Method

### Participants

**Fig. 1:**
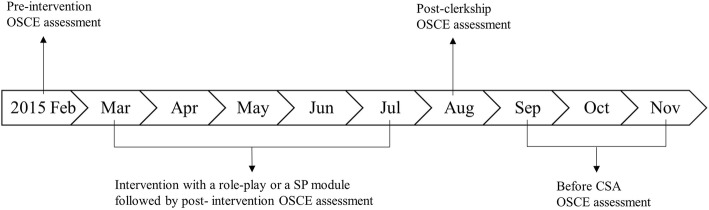
Timeline of the study regarding the OSCE assessment. OSCE; Objective Structured Clinical Examination . SP; Standardized Patient. CSA; Clinical Skill Assessment

We enrolled 113 participants which was the full year cohort of fourth-year medical students attending a major medical school in Korea during 2015. The fourth-year medical school curriculum involves clinical rotations, including a family medicine clerkship from March through July. For regular clinical clerkships, students were divided into fourteen small groups (six to nine students per group). This study was conducted during one week of the family medicine clerkship in which smoking-cessation counseling teaching is an integral part. Figure 1 shows the timeline of this study. Both enrollment and retainment rate were 100% since all students participated the study and completed all the assessments.

Students gave consent to enter the study before randomization. Students were allocated either to a standardized patients (SPs) group or to a role-play group on the basis of the group randomization by the order of clinical clerkship rotation [25, 26]. Random digit number was produced using Excel® software program by the third person. Each group completed the intervention module predetermined by randomization.

To achieve a power of 0.8 with a two-sided significance level of 5% for a minimum effect size of 0.6 with respect to OSCE scores, a required sample size of 45 for each group was calculated. The effect size was based on the pooled standard deviation of 8.0 estimated by an interim analysis of two intervention groups. As one item score was 4.0 from full marks of 100 points for the OSCE station with 25 checklist items, effect size of 0.6 was equivalent to around the score of 1.2 items.

### Pre-intervention

Students’ tobacco-cessation counseling skills were evaluated as a pre-intervention OSCE score to determine baseline performance. As part of the standard curriculum, medical students were assessed according to their clinical skills by the OSCE involving twelve SP encounters in February 2015, a month before the regular clinical clerkships. The scenarios and marking schemes for each OSCE station were developed and agreed by consensus of local experts. Face validity was assessed by the panels developing the stations. Tobacco-cessation counseling occurred in one of the twelve SP encounters. Students were given ten minutes to evaluate and counsel a smoking patient. Prior to the intervention during the family-medicine clerkship, we measured students’ baseline knowledge of smoking by written test and their self-confidence to counsel their own smoker-patients by self-administered questionnaire on a ten-point Likert scale from 1 to 10 (from 1 = extremely inconfident to 10 = extremely confident in smoking cessation counseling). The written test was the questionnaire of 43 items to assess medical students’ knowledge of tobacco cessation and treatment for nicotine addiction, which was developed according to previous related literatures [27, 28]. It consisted of four knowledge sections, i.e., smoking epidemiology (4 items), health risks of smoking (19 items), benefits of tobacco cessation (3 items), and treatment of nicotine addiction (17 items). When reliability of the cognitive competence questionnaires was determined by the Rasch model, it was 0.97 for pre-test and 0.95 for post-test, respectively. Local experts in smoking cessation and primary care physicians in the university hospital assessed its face validity as good. To prepare for the intervention, students were instructed to discuss and develop an OSCE checklist in their group. This checklist was utilized to provide each student doctor feedback on his or her performance by a SP or a student observer in each module play.

### Peer role-play module

Students worked in groups of three and alternatively role-played the doctor, the patient, and the observer. Prior to the role-play, each group was instructed to develop a scenario for smoking cessation. Role-play involved a ten-minute encounter followed by five minutes of feedback from the observer of each role-play episode. A group-developed OSCE checklist was used to rate the performance and provide feedback. One cycle of a role-play took fifteen minutes, which consisted of the ten-minute encounter and five minutes of peer feedback. Three cycles of a role-play comprised one round. The first round was conducted using a scenario developed by each group, and then students exchanged the scenario between groups for the second round. Groups of three students proceeded to role-play through three rounds of the cycle as a whole. The total intervention lasted three-and-half hours and was supervised by faculty staff.

### SP module

In this module, two experienced actors were trained as SPs to portray a smoker with a willingness to quit smoking. SPs received in-depth training by the faculty staff for about two hours. Students in the SP module did an interview with one female SP and one male SP. Each student was asked to privately counsel a SP in an examination room for ten minutes. Other students observed the interview remotely through cameras placed in the room. After the encounter, every SP provided five minutes of direct oral feedback to the student. Each student had three encounters with a SP and received feedback for each. The total intervention lasted three-and-half hours and was supervised by faculty staff, as for the role-play module.

### Post-intervention

At the end of the family medicine clerkship, following the intervention, students’ smoking cessation intervention skills were assessed again with a post-intervention OSCE score. The post-intervention OSCE consisted of a single SP case. This mini-OSCE was used to assess post-intervention students’ smoking cessation counseling skills. Students were given ten minutes to evaluate and counsel a smoking patient. Same as the pre-intervention, students were administered a post-intervention evaluation for smoking knowledge and self-confidence levels. Also, the survey gauged the students’ satisfaction with each module.

After finishing the regular clinical clerkship, students’ counseling skills were assessed through a mini-OSCE that evaluated knowledge of a clinical scenario other than smoking cessation. This accounted for a post-clerkship OSCE score that represented the short-term effects of the training. A new scenario used in the assessment was different in terms of the SP’s motivational stage toward smoking cessation compared with the post-intervention OSCE scenario, in which the SP was willing to quit smoking. The purpose of the new scenario was to evaluate the transferability of students’ counseling ability as well as the short-term effect of the intervention.

Students did take the Clinical Skill Assessment (CSA) of Korean Medical Licensing Examination (KMLE) between September and November. Prior to taking the KMLE-CSA, students were evaluated via a mini-OSCE on smoking-cessation counseling in such a way as to be representative of the long-term effects of the training. All OSCE and mini-OSCEs on tobacco-cessation counseling were scored using a 17-item behavioral checklist with dichotomous scoring and an 8-item behavioral checklist with a seven-point scale. SPs conducted standardized assessment of students’ performances.

### Data analysis

Descriptive statistics were used for the student demographic data. To measure the effects of the two modules on student OSCE scores mean scores for the OSCE pre- and post-intervention were compared using a paired t-test. The total OSCE score consisted of four components: patient satisfaction (PS), history taking (HT), patient education (PE), and patient-physician interaction (PPI), of which we compared the difference between pre- and post-intervention with a paired t-test. Student’s t-test was used to compare the OSCE scores, self-confidence ratings, and smoking-knowledge test scores between the SP module and the role-play module groups. To examine the retention effect of the training, pre-intervention OSCE scores, post-intervention OSCE scores, post-clerkship OSCE scores, and OSCE scores before the KMLE-CSA were analyzed using repeated-measures analysis of variance with post-hoc analysis by Bonferroni’s method. The assumption of sphericity and equality of covariance matrices were satisfied. Effect sizes were calculated using Cohen’s *d*. *P*-values less than 0.05 were considered statistically significant. All analyses were conducted using SPSS, version 21.0 (SPSS Inc., Chicago, IL).

## Results

A total of 113 medical students in their fourth year participated in the study. Average student age was 26.2 years. There were no significant percentage differences in age, sex, and proportion of smokers between the SP module group and the role-play group (Table 1).

**Table 1:** Student demographics (*N* = 113)

	Students taking the standardized patient (SP) module^a^ (*N* = 57)	Students taking the peer role-play module^b^ (*N* = 56)	Total (N = 113)	P-value*
Age	26.5 ± 3.0	25.9 ± 2.1	26.2 ± 2.6	0.23
Sex (male, %)	36 (63.2)	35 (62.5)	71 (62.8)	0.94
Smoking status (non-smoker, %)	48 (84.2)	48 (85.7)	96 (85.0)	0.74
Alcohol consumption (drinker, %)	50 (87.7)	49 (87.5)	99 (87.6)	0.12

Values are presented as mean ± SD or number (%)

^a^One-to-one communication training with direct oral feedback from the standardized patient

^b^Training with peer role-playing and mutual feedback

*Student t-test between the case group and the control group

**Fig. 2:**
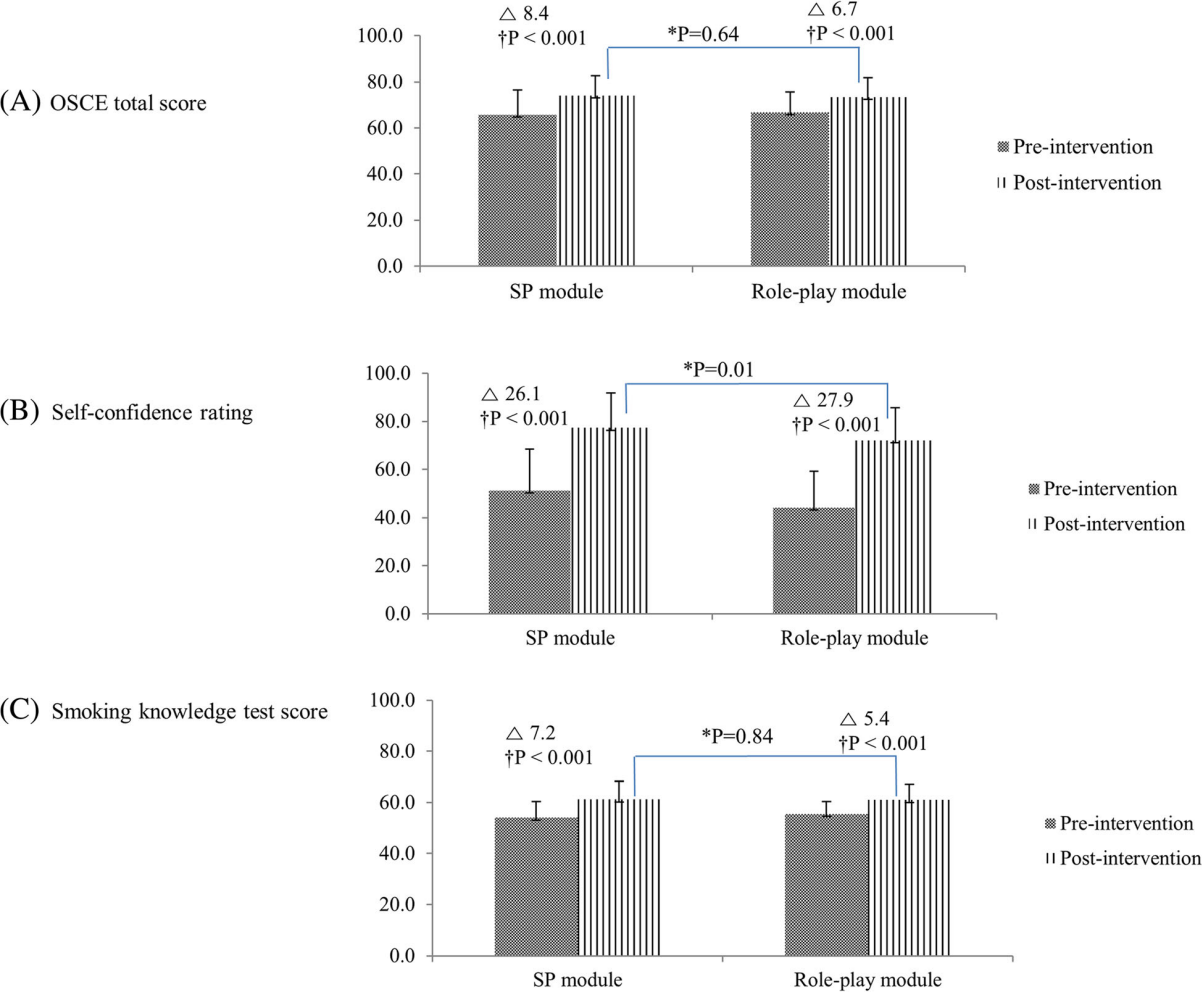
Comparison of pre-intervention and post-intervention scores for two groups. (**a**)OSCE total score. (**b**)Self-confidence rating. (**c**)Smoking knowledge test score. SP; Standardized Patient, OSCE; Objective Structured Clinical Examination. *P-value by independent t-test between a standardized patient module and a role play module. †P-value by paired t-test between pre-intervention and post-intervention scores

Figure. 2A shows the differential effects of the SP module and the role-play module on the students’ OSCE scores. In the SP module group, the post-intervention OSCE scores increased significantly (Cohen’s *d* 0.87, *p* < 0.001) compared with the pre-intervention OSCE scores, along with a significant increase in the HT, PE, and PPI components (component data not shown in the figure). In the role-play group, post-intervention OSCE scores were significantly higher than the pre-intervention scores (*d* 0.77, *p* < 0.001); the HT and PE components increased significantly. After their training, students’ self-confidence and smoking-knowledge scores also increased significantly, regardless of module type (all *p* < 0.001) (Fig. 2B, C).

In the post-intervention comparisons, post-intervention OSCE scores and smoking-knowledge scores were not statistically different between two groups. Student post-intervention self-confidence levels were significantly higher in the SP group than in the role-play group (*d* 0.37, *p* = 0.01), with higher baseline self-confidence levels in the SP group also. Students’ satisfaction levels on each module were equivalent between two modules (Table 2).

**Table 2:** OSCE scores, self-confidence ratings, knowledge-test scores, and student satisfaction with each module: Comparison of the two groups

	Pre-intervention			Post-intervention		
	Standardized patient	Role-play	P-value*	Standardized patient	Role-play	P-value*
OSCE scores						
Patient satisfaction	66.0 ± 17.3	66.4 ± 17.1	0.89	69.1 ± 15.6	68.2 ± 17.0	0.77
History taking	77.6 ± 17.8	80.4 ± 11.0	0.32	87.5 ± 10.7	86.9 ± 8.5	0.74
Patient education	52.2 ± 18.6	50.7 ± 18.1	0.66	64.0 ± 16.7	62.1 ± 16.5	0.53
Patient-physician interaction	65.8 ± 10.3	67.6 ± 10.1	0.35	69.1 ± 10.5	69.6 ± 10.2	0.79
Total score	65.7 ± 10.7	66.7 ± 8.9	0.58	74.1 ± 8.6	73.4 ± 8.4	0.64
Self-confidence rating	51.1 ± 17.4	44.2 ± 15.0	0.02	77.3 ± 14.6	72.1 ± 13.5	0.01
Knowledge test score	54.0 ± 6.4	55.5 ± 4.9	0.21	61.2 ± 7.0	61.0 ± 6.1	0.84
Student satisfaction				81.0 ± 14.9	77.4 ± 14.7	0.10

OSCE; Objective Structured Clinical Examination

Values are presented as mean ± SD

*Student t-test of OSCE scores between the two groups

**Fig. 3:**
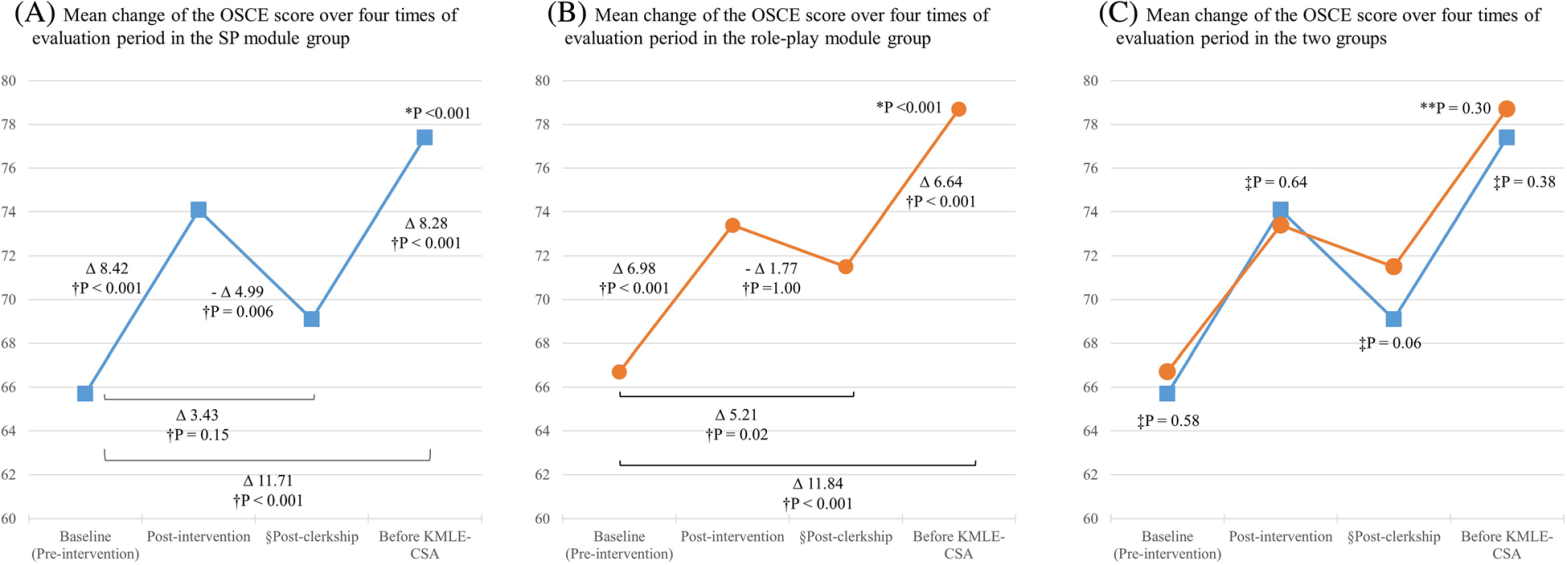
Mean change of the OSCE score over four times of evaluation period in the SP group and the role-play group. (**a**)Mean change of the OSCE score over four times of evaluation period in the SP module group. (**b**)Mean change of the OSCE score over four times of evaluation period in the role-play module group. (**c**)Mean change of the OSCE score over four times of evaluation period in the two groups. †P-value by post hoc comparisons within each group for repeated measures with Bonferroni correction. *P-value by repeated measures analysis of variance. ‡P-value by post hoc comparisons between groups for repeated measures with Bonferroni correction. **P-value of group-by-time interaction by repeated measures analysis of variance. §Assessment with a new scenario which is different in terms of the SP’s motivational stage toward smoking cessation. OSCE; Objective Structured Clinical Examination, SP; Standardized Patient, KMLE; Korean Medical Licensing Examination, CSA; Clinical Skill Assessment

Figure 3 shows the long-term retention effect as well as the short-term effect of each module. Among the four assessments—pre-intervention OSCE, post-intervention OSCE, post-clerkship OSCE, and OSCE before the KMLE-CSA—students’ OSCE scores were highest before the KMLE-CSA in both groups (*p* < 0.001 in both groups). In the SP group, the post-clerkship OSCE score for the new scenario was higher than the pre-intervention score without significance (*d* 0.41, *p* = 0.15) (Fig. 3A). In the role-play group, the post-clerkship OSCE score for the new scenario was higher than the pre-intervention score (*d* 0.62, *p* = 0.02) (Fig. 3B). Figure 3C revealed that there were no significant differences between the two modules for short-term or long-term retention effects (*d* 0.35, *p* = 0.06 in post-clerkship scores; *d* 0.16, *p* = 0.38 in scores before the KMLE-CSA). There was no significance of group-by-time interaction in the OSCE score by repeated-measures ANOVA (*p* = 0.30).

## Discussion

In this study, undergraduate medical students’ OSCE scores for tobacco-cessation counseling increased significantly after education by both the SP and peer role-play modules. The HT and PE components of the OSCE scores improved significantly after both modules, while the PPI component improved significantly after the SP module only. Students improved significantly in smoking knowledge according to written test scores as well as in self-confidence after both modules. Although there were no differences between the two modules with regard to post-intervention OSCE scores or post-intervention smoking-knowledge scores, the SP module for tobacco-cessation education yielded better results for student self-confidence than the peer role-play module. Students were equally satisfied with their education modules. The short-term and long-term effects on retention of tobacco-cessation education were seen from both modules after two to eight months.

For successful smoking cessation, individualized smoking-cessation counseling and pharmacological intervention are required based on different behavioral stages of smoking cessation [29]. To achieve successful cessation, appropriate education and training are needed at the undergraduate level; thus, physicians should be well-prepared in terms of both smoking knowledge and interview skills [30]. There are several approaches to smoking-cessation training, which can be classified into traditional methods, such as didactic lectures or handouts, and interactive or practical methods, such as case reports, problem-based learning, role-play, and interviewing a standardized patient [19, 20, 30, 31].

The two modules in our study utilized active learning or practical skills for smoking-cessation counseling training. A previous study demonstrated that passive learning methods, such as didactic lectures, as well as active-learning methods, can help medical students learn the basic principles of smoking-cessation counseling [32–34]. In our study, medical students’ smoking-cessation knowledge increased after both the role-play module and the SP module. Since a lack of perceived self-confidence has been considered to be one of the major barriers in tobacco-cessation counseling, educational modules that improve medical student self-confidence have been increasingly recommended [35]. As for smoking-cessation counseling education worldwide, the percentage of schools that use an interactive method or patient-centered counseling was reported to be low—only one third of medical schools [31]. Also, practical-skills training for smoking-cessation counseling was reported to be insufficient in the UK [19]. Interactive learning methods, rather than didactic lectures, have been reported to improve the communication skills and self-confidence of medical students during their tobacco-cessation counseling [20, 22, 30]. Students in our study showed an increase in self-confidence after both modules, although it was higher in students who were educated with the SP module than the role-play module. Although students in the SP module group felt more confident in smoking cessation counseling, however, this self-perceived confidence didn’t impact on learning outcomes such as OSCE scores.

Teaching methods involving role-play or SPs have both strengths and weaknesses. Using SPs to teach and evaluate communication skills provides medical students an opportunity to practice skills in a safe and relatively unthreatening environment [36]. Students can repeatedly prepare for an actual clinical problem through SP encounters and be objectively assessed on their communication skills [37, 38]. Also, using SPs can achieve uniformity in case scenarios, in addition to the potential benefits of receiving SP feedback [39]. However, organizing SP modules is costly and complex [40]. Compared with the SP module, peer role-play requires few resources but may not be taken seriously by students [21]. In the peer-role play module, students may feel a greater burden of the training as a result of developing and learning a scenario and exchanging the role of doctor and patient among each other. However, students can experience the role of a patient in the peer role-play module, but not in the SP module. Playing the role of a patient may help students improve their patient counseling skills and deepen their education [41]. The potential effectiveness of peer feedback with role-play for developing smoking-cessation skills has been reported to be at least equal to the SP module [20, 42]. In this study, medical students who were provided with peer role-play practice were found to improve significantly in their self-confidence and knowledge of smoking cessation. This suggests that each medical school can choose an appropriate active-learning method for teaching smoking-cessation counseling. For example, if there is a sufficient budget, the SP module may be desirable; however, for smaller budgets, the role-play module can also be an effective training tool for student smoking-cessation counseling. For practical purposes, it was necessary to evaluate how the two modules yielded an increase in the OSCE score of smoking-cessation counseling, since medical students must complete the OSCE for their medical license examination. One study demonstrated that education using interactive teaching methods, including SP or role-play, did not yield higher OSCE scores than the control group [30]. However, in other studies that evaluated the role-play method, the educational effect was reflected in the OSCE score of dental student smoking-cessation counseling [42, 43]. In this study, the post-intervention OSCE scores of medical students were significantly higher than the pre-intervention scores for both modules, and they reflect the short-term effect of educational training. Comparisons of post-intervention OSCE scores in each module were not significantly different. However, it is noteworthy that both modules showed a significant increase in the HT and PE components of the OSCE score, as well as an increase in the PPI after the SP module only. The differential effects of these components reflect the impact of interactive counseling education on medical students. Meanwhile, the post-clerkship OSCE scores for the scenario that was different from the other three OSCE were higher than in the pre-intervention scores but lower than the post-intervention scores. The scores before the KMLE-CSA, the final OSCE scores, were the highest among the four evaluation periods. This may be attributable to the effect of students’ self-directed learning ahead of the KMLE with repeated role-play and peer feedback, in addition to the long-term retention effect of education in each module. Also, there is a possibility that an information-sharing effect among students may exist. In every assessment over the four time periods, students were tested sequentially and they knew that those OSCE scores were being graded. This explains the potential impact of the students’ goal orientation as well as the self-study other than the module effect on the final scores around the KMLE assessment.

One limitation of this study may be its limited generalizability, because it was conducted at a single medical school. The assessment using a single OSCE case may further limit the generalizability when considering variations in smoking patients’ readiness to quit. Also, the different timing of the assessments for each group may have affected the objective measurement of OSCE scores due to information-sharing behavior of the students. The clerkship experience during the semester may have influenced students’ self-assessment of their self-confidence. In addition, allocation concealment or blinding could not be performed due to the nature of the education course and the study design. Therefore, students in the SP module group could peer-role play or vice versa. All students would have done self-directed learning for smoking cessation counseling in the end to prepare for the KMLE. This could illustrate another consequence of bias. Finally, since the students of role-play module group developed their own scenario, they all played patients at a different stage of change compared to the students of SP group. That would also bias the results.

Despite these limitations, our findings highlight the effectiveness of smoking-cessation counseling training in the medical school curriculum. These findings provide some evidence that peer role-play may be an equivalent educational method in terms of smoking cessation counseling skills and knowledge compared to the SP method. Our study suggests that it is necessary for medical schools to choose one or the other educational module for teaching smoking-cessation counseling, considering cost and the effectiveness of a module.

## Conclusions

Appropriate smoking cessation counseling education is required at the undergraduate level for physicians to have counseling skills. This study not only compared the role play module to SP module in terms of smoking knowledge test score, self confidence level, and OSCE score, but also analyzed the long-term effects of education. Both modules were effective in increasing the OSCE score, self-confidence and knowledge score of the smoking cessation counseling. Above all, the role play module was equivalent in several aspects to the SP module. It will be important to compare the two methods for teaching smoking cessation counseling according to educational conditions.

## Data Availability

The datasets and materials can be obtained from the corresponding author upon reasonable request.

## Abbreviations

CSA: Clinical Skill Assessment
HT: History Taking
KMLE: Korean Medical Licensing Examination
OSCE: Objective Structured Clinical Examination
PE: Patient Education
PPI: Patient-Physician Interaction
PS: Patient Satisfaction
SP: Standardized Patients
